# Stochastic Model of Solvent Exchange in the First Coordination Shell of Aqua Ions

**DOI:** 10.1021/acs.jctc.2c00181

**Published:** 2022-04-26

**Authors:** Luca Sagresti, Lorenzo Peri, Giacomo Ceccarelli, Giuseppe Brancato

**Affiliations:** † Scuola Normale Superiore, Piazza Dei Cavalieri 7, I-56126 Pisa, Italy; ‡ Istituto Nazionale di Fisica Nucleare (INFN), Largo Pontecorvo 3, I-56127 Pisa, Italy; § Dipartimento di Fisica, Università di Pisa, Largo Bruno Pontecorvo 3, I-56127 Pisa, Italy; ∥ Consorzio Interuniversitario per Lo Sviluppo Dei Sistemi a Grande Interfase (CSGI), Via Della Lastruccia 3, I-50019 Sesto Fiorentino (FI), Italy

## Abstract

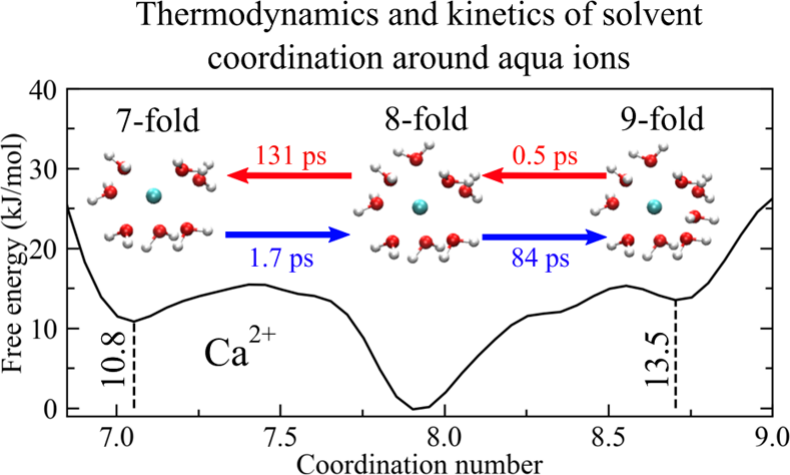

Ion microsolvation is a basic, yet fundamental, process of ionic solutions underlying many relevant phenomena in either biological or nanotechnological applications, such as solvent reorganization energy, ion transport, catalytic activity, and so on. As a consequence, it is a topic of extensive investigations by various experimental techniques, ranging from X-ray diffraction to NMR relaxation and from calorimetry to vibrational spectroscopy, and theoretical approaches, especially those based on molecular dynamics (MD) simulations. The conventional microscopic view of ion solvation is usually provided by a “static” cluster model representing the first ion–solvent coordination shell. Despite the merits of such a simple model, however, ion coordination in solution should be better regarded as a complex population of dynamically interchanging molecular configurations. Such a more comprehensive view is more subtle to characterize and often elusive to standard approaches. In this work, we report on an effective computational strategy aiming at providing a detailed picture of solvent coordination and exchange around aqua ions, thus including the main structural, thermodynamic, and dynamic properties of ion microsolvation, such as the most probable first-shell complex structures, the corresponding free energies, the interchanging energy barriers, and the solvent-exchange rates. Assuming the solvent coordination number as an effective reaction coordinate and combining MD simulations with enhanced sampling and master-equation approaches, we propose a stochastic model suitable for properly describing, at the same time, the thermodynamics and kinetics of ion–water coordination. The model is successfully tested toward various divalent ions (Ca^2+^, Zn^2+^, Hg^2+^, and Cd^2+^) in aqueous solution, considering also the case of a high ionic concentration. Results show a very good agreement with those issuing from brute-force MD simulations, when available, and support the reliable prediction of rare ion–water complexes and slow water exchange rates not easily accessible to usual computational methods.

## 1 Introduction

**Figure 1:**
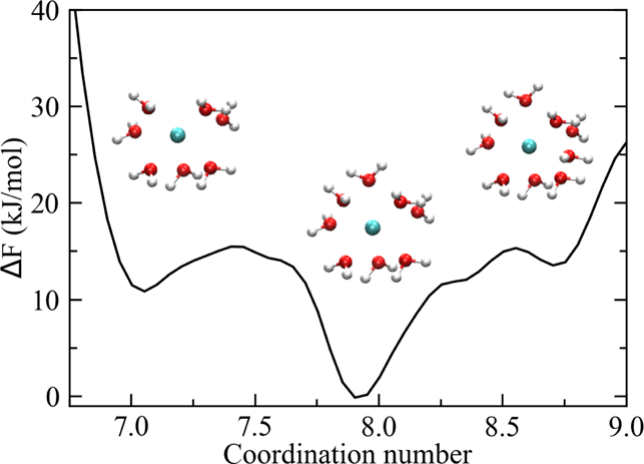
Free-energy change (Δ*F*) as a function of the (continuous) solvent coordination number of Ca^2+^ in aqueous solution, obtained according to the method of ref (22) (see text for details). Representative ion–water complexes with seven, eight, and ninefold coordinations are depicted as insets.

Ion–water coordination and exchange play a primary role in many physical, chemical, biological, and technological processes, such as aqueous solution structures,^1^ catalytic activity,^2^ ion transport,^3^ materials design,^4^ and so on. Therefore, from the elucidation of the detailed structural and dynamic features of ion microsolvation, a better comprehension of various complex phenomena may follow, such as the water exchange mechanism in the first hydration shell, the solvent reorganization energy between ion redox couples,^5^ or the electrostriction effect in ionic solutions.^6^ Besides standard laboratory techniques, such as X-ray and neutron diffraction spectroscopy,^7,8^ NMR^9^ and dielectric^10^ relaxation measurements, thermodynamic measurements,^11,12^ and vibrational spectroscopy,^13^ theoretical approaches rooted into molecular dynamics (MD) simulations, based on either force-field^14,15^ or quantum-mechanical^16−19^ potentials, proved very valuable in providing detailed information about ion coordination and solvent exchange. Yet, in most cases, ion coordination is described through simple structural parameters, such as the average ion–water distance or the average coordination number, which is often insufficient to understand the variable behavior the ions show in many circumstances, such as the debated “gadolinium break”^20^ or the nonlinear solvent response induced by redox reactions.^5^ Thankfully, a more comprehensive picture can be gained by the free-energy profile of ion coordination^21,22^ (see, e.g., Figure 1), as seen in the framework of the “quasi-chemical theory” by Pratt and co-workers.^23,24^ Indeed, for a given ion, the ion-coordination free-energy landscape nicely illustrates the most accessible ion–water configurations, provides the energy difference among various complexes, and estimates the energy barrier to be overcome during water exchange events, that is, the energy cost for losing or acquiring a water molecule in the first hydration shell. Moreover, inspection of the free-energy profile reveals whether the water exchange mechanism follows preferentially a dissociative or an associative pathway.^25^ Hence, such free-energy landscapes do enrich significantly our understanding of ion coordination in aqueous solution by providing a further dimension to our physico-chemical knowledge of ion solvation. In this context, we recently proposed a metadynamics (meta-MD)^26^-based method to obtain accurate free-energy profiles of ion coordination in aqueous solutions.^22^ The method addresses the computational problem concerning the collection of an extended molecular sampling as needed for the evaluation of the free-energy contribution to ion–water coordination. According to this approach, a rather complete structural and thermodynamic picture of ion coordination can be obtained through relatively short meta-MD simulations, as illustrated by application to a variety of mono-, di-, and trivalent ions in aqueous solutions.^22,27^ One advantage of this method is that the “reaction coordinate,” which corresponds to the solvent coordination number, is not biased toward a specific water exchange pathway or mechanism.

In addition to the detailed structural and thermodynamic characteristics of ion microsolvation, however, it is also important to assess the water exchange dynamics in the first coordination shell since this is closely related to the kinetics of various molecular processes, such as ion transport in narrow protein channels or ion-catalyzed reactions. Water exchange rates, as usually determined by NMR experiments,^28,29^ span several orders of magnitude (i.e., from 10^–12^ to >10^6^ s)^2,29^ and, therefore, are generally not accessible to standard MD simulations. Extensive theoretical work was carried out to develop effective approaches to study exchange rates, especially within the framework of the transition state theory (TST).^30−32^ Among others, two methodologies emerged as the most frequently adopted: the reactive flux^33^ and the transition path sampling^34^ techniques, though the former could be biased by the choice of the specific reaction coordinate and the latter typically requires a substantial computational effort. As an alternative to TST-based methods, however, reaction rates can also be determined from a master-equation approach that exploits the concept of mean first-passage time (MFPT).^35^

In this work, starting from the notion of free-energy landscape of ion–water coordination^22^ as seen above, we propose an effective computational strategy to estimate ion coordination and water exchange rates in the first solvation shell around aqua ions. In particular, the exchange rates are determined in terms of MFPTs between different ion–water configurations, as obtained by a purposely developed stochastic model. The model, which is based on the one-dimensional Fokker–Planck (FP) equation, assumes that the exchange process is Markovian, given a suitable discretized reaction coordinate (i.e., water coordination number, *s*). In addition to the free-energy function Δ*F*(*s*), the key ingredient of the stochastic model is represented by the position-dependent diffusion coefficient, *D*(*s*). Here, *D*(*s*) was evaluated following the method proposed by Hummer,^36^ which is based on the calculation of the transition rate matrix assuming detailed balance at equilibrium. The present kinetic model was successfully tested against results issuing from direct MD simulations by considering Ca^2+^, Zn^2+^, Hg^2+^, and Cd^2+^ in aqueous solution as test cases. While most tests were performed on dilute solutions, in one case, we also showed the application to a high molar concentration. Besides, we devised an effective methodology to address the case of rare exchange events not accessible to standard MD, thus allowing the reliable prediction of slow rates at an affordable computational cost. As a further important result obtained in this study, we showed, through the application of a committor analysis, that the water coordination number is not only a convenient and intuitive collective variable for describing ion–water coordination but also a physically sound “reaction coordinate” for the exchange process.^37,38^

## 2 Theory and Methods

### 2.1 Free Energy of Ion Coordination

In this work, similar to previous studies,^21,23^ we made the assumption of describing the first hydration shell around a given ion in terms of the water coordination number, hereafter denoted as *s*, as an effective collective variable for the solvation process. The free energy of ion coordination in aqueous solution, Δ*F*(*s*), was conveniently expressed as a function of the solvent coordination number (see, e.g., Figure 1), which was defined as a continuous parameter according to the method described in ref (22). For a given ion–water molecular configuration, the coordination number is, then, expressed as
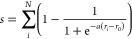
1where the sum is extended over the total number, *N*, of solvent molecules, *r*_*i*_ is the ion–oxygen distance of the *i*-th water molecule, and *r*_0_ and *a* are, respectively, the ion–oxygen cutoff distance and the parameter of the switching (exponential) function that smoothly goes from 1 to 0 across *r*_0_ (see ref (22) for more details). In particular, for each ion considered, the parameter *r*_0_ was set to the distance of the well minimum following the first peak of the corresponding ion–oxygen radial distribution function (i.e., *r*_0_ was within the range of 3.0–3.4 Å, Figure S1). This choice was based on the idea of including all solvent molecules in the first coordination shell. The smoothing parameter *a* was set in all simulations to 4.0 Å^–1^ according to some tests performed in our previous work.^22^ From extended samplings of the configurational space, as obtained by either standard MD or meta-MD simulations, the free-energy landscape of ion coordination, Δ*F*(*s*), was evaluated for all cations under scrutiny in this study (Figure S2). Note that the statistical error affecting Δ*F*(*s*) can be made systematically small by extending the configurational sampling. Then, according to the present method, accurate estimates of Δ*F*(*s*) can be obtained at an affordable computational cost, provided that a reliable ion–water interaction potential is employed.

### 2.2 Water Exchange Dynamics

A stochastic kinetic model was developed to describe the water exchange dynamics in the first solvation shell, that is to estimate the water exchange rates between different ion–water configurations. Assuming the dynamical process is Markovian for a proper coarse-grained discretization of the reaction coordinate (i.e., ion–water coordination number), a kinetic model based on the one-dimensional FP equation, also known as the Smoluchowski equation, was developed^39^
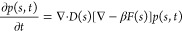
2where *p*(*s*,*t*) is the time-dependent probability distribution density, β = (*k*_B_*T*)^−1^ is the Boltzmann factor (i.e., the inverse of the Boltzmann constant, *k*_B_, times the temperature, *T*) and *D*(*s*) is the position-dependent diffusion coefficient of *s*. As an alternative approach, the water exchange dynamics can be equivalently described by the (overdamped) Langevin equation which expresses the (stochastic) equation of motion of coordinate *s*
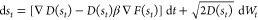
3where *s*_*t*_ ≡ *s*(*t*) and d*W*_*t*_ is the Wiener process. In the latter case, transition rates are obtained by averaging the arrival times over multiple Langevin dynamics (LD) simulations. Along with *F*(*s*), *D*(*s*) is the second most important ingredient needed to fully define the kinetic model, and it was evaluated as described in the following.

### 2.3 MFPT

Water exchange rates were evaluated in terms of MFPTs between different coordination number configurations. Note that MFPTs can be evaluated in different ways. In particular, if the coordination space is accessible to standard MD simulations, MFPTs can be directly obtained from the analysis of the corresponding trajectories. Otherwise, in cases of rare transitions, MFPTs can be evaluated from the present kinetic model by solving numerically either the FP equation or the corresponding backward Kolmogorov–Chapman equation.^40^ Exploiting the same kinetic model, MFPTs can also be obtained from the equivalent LD simulations.

**Figure 2:**
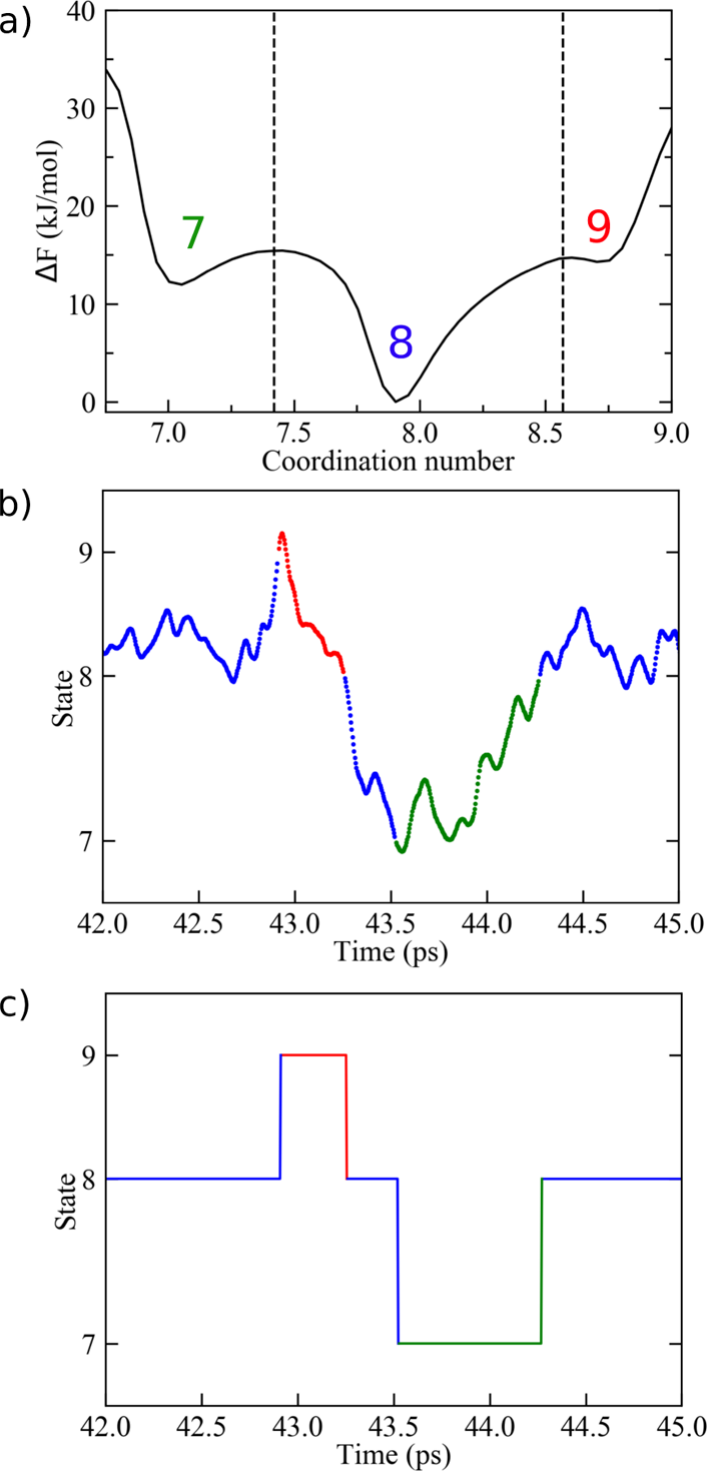
(a) Partitioning (dashed lines) of the coordination number space in contiguous regions representing different metastable states (i.e., 7, 8, and 9) on the basis of the free-energy profile of Ca^2+^ in water. (b) Trajectory of the *s* coordinate (solid line) during a given time interval of the Ca^2+^ MD simulation. At each time step, the system is assigned to one of the possible coordination states according to the history-based method described in the text. Green, blue, and red colors correspond to states 7, 8, and 9, respectively. (c) Same trajectory, after the assignment, is converted into a discrete number representation (i.e., coordination state number). Note that the overall residence time, τ_*i*_, of the system in state *s*_*i*_ is given by the sum of all time intervals assigned to *s*_*i*_.

First, on the basis of the computed free-energy profile *F*(*s*) for a given ion–water system, the coordination number space was partitioned into a discrete number of consecutive coordination states, *s*_*i*_ (i.e., different regions of *s*), in correspondence to the free-energy local minima, each one limited by adjacent energy barriers (Figure 2a). Accordingly, the MFPT was defined as the average time spent by the system in each coordination state before jumping to a different one. Since ions generally showed three or more main coordination states, the MFPT, τ_*ij*_, to jump from a given state *s*_*i*_ to an adjacent state *s*_*j*_ (with *j* = *i* ± 1) was defined as the ratio between the overall residence time in the *i*-th state (τ_*i*_) and the number of *i* → *j* state transitions (*n*_*ij*_), that is, τ_*ij*_ = τ_*i*_/*n*_*ij*_.

From standard MD (or LD) simulations, the τ_*ij*_'s were obtained by initially assigning each configuration of the trajectory to a unique coordination state according to a history-based algorithm: each configuration sampled at a given time *t* was assigned to state *s*_*i*_ if, at a previous time *t*′ with *t*′ < *t*, the coordinate *s*(*t*) crossed the local minimum configuration of *s*_*i*_ (see Figure 2b,c). This choice prevented the counting of spurious jumps between states (i.e., fast barrier recrossings) while, at the same time, it ensured that transitions occurred only between adjacent coordination states. To validate this procedure, we compared the population of states (*s*_*i*_) issuing from such a history-based method with the one obtained by mapping directly each configuration of the MD trajectory onto state *s*_*i*_ corresponding to the partition visited. As a result, no significant differences appeared (Figure S3). While other time-based criteria were also tested for assigning MD configurations to a given state *s*_*i*_, as for example, the use of a “minimum residence time,” the procedure described above appeared as the most satisfactory for treating the ion–water coordination dynamics. Also, note that the present methodology is conceptually similar to the one used by Milestoning.^41^

Within the framework of our stochastic model (i.e., a birth–death process where transitions are allowed only between adjacent coordination states), the conditional MFPT between states *s*_*i*_ and *s*_*j*_ (starting from *s*_*i*_ at *t* = 0) can be expressed as^42^
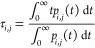
4where *p*_*i*,*j*_(*t*) is the conditional probability density that the system reaches *s*_*j*_ at time *t* upon starting from *s*_*i*_ at time zero *p*_*i*,*j*_(*t*) = *p*(*s*_*j*_,*t*|*s*_*i*_,0). The integral corresponds to the “splitting” probability to end up in *s*_*j*_ (see, e.g., ref (42)). Eq 4 can be solved, once the probability density *p*(*s*,*t*) is known, by assuming an adsorbing (at the ending state) and a reflecting (preceding the starting state) boundary condition. Among other methods, the latter can be integrated numerically with the Crank–Nicolson scheme.^43^ As a more convenient alternative, the MFPT can be also obtained directly from the adjoint equation of the FP (i.e., also known as backward Kolmogorov–Chapman equation) by solving the integral (see, e.g., ref (40))
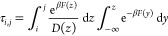
5

Furthermore, an approximate well-known result for τ_*i*,*j*_ is provided by the Kramers theory,^44^ which, in the limit of an overdamped dynamics, gives the compact formula^35^
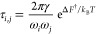
6where γ is the friction coefficient (γ = *k*_B_*T*/*D*), ω_*i*_ and ω_*j*_ are the angular frequencies at the well bottom of *s*_*i*_ and *s*_*j*_, respectively, and Δ*F*^†^ is the energy barrier for the *i* → *j* transition. The angular frequency can be approximated as . In this work, the Kramers’ MFPT was also evaluated, for the sake of comparison, assuming that the constant coefficient *D* was given by , with *D*(*i*) and *D*(*b*) being the diffusion at the bottom of *s*_*i*_ and at the peak of the barrier, respectively. Statistical errors of the τs were estimated from the exponential fit of the distribution of the arrival times, as obtained from the MD simulations. In the case of the stochastic approach, the same errors were estimated from the corresponding uncertainty of the diffusion coefficient (*D* ± δ*D*) as described in the next section.

### 2.4 Position-Dependent Diffusion Coefficient

Following the method proposed by Hummer,^36^ a position-dependent diffusion coefficient along the coordination number, *D*(*s*), was obtained from a master-equation approach upon partitioning evenly the configurational space into *N* non-overlapping regions of width Δ*s*
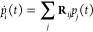
7where *p*_*i*_(*t*) is the probability of being in region *i* at time *t* and **R**_*ij*_ is the transition rate matrix with constant coefficients. The solution of this equation can be expressed in terms of the propagator^36^
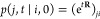
8which expresses the probability of finding coordinate *s* within the region *j* at time *t*, after starting at *i* at time *t* = 0. The rate matrix **R**_*ji*_ is related to the position-dependent diffusion coefficient through the equation
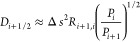
9where *D*_*i*+1/2_ represents the arithmetic mean [*D*(*s*_*i*_) + *D*(*s*_*i*+1_)]/2, Δ*s* is the discretization step, and *P*_*i*_ is the equilibrium population of the *i*-th region (note that *P*_*i*_ = exp[−Δ*F*(*s*_*i*_)/*k*_B_*T*] and is readily obtained from MD or meta-MD simulations). In practice, the propagator (eq 8) is constructed from the observed local transitions (from *i* to *j*) during the MD simulations, given a fixed lag time Δ*t*. The rate matrix **R**_*ji*_ is obtained through the routine linalg.logm^45^ of Scipy (v. 1.5.4), and as a result, the position-dependent diffusion coefficient, *D*(*s*), was determined from eq 9. Note that the discrete regions do not correspond to the previous coordination states, but they are the result of a finer partitioning of the coordination number space.

In the case of high free-energy barriers and rare transition events, our approach takes advantage of the molecular sampling obtained during the meta-MD simulations to extract starting configurations throughout all coordinate spaces in order to run short MD runs (250 ps), aiming at determining the required local transition probabilities used to define the propagator, (e^*t***R**^)_*ji*_. For each starting configuration, many MD replicas (>100) were carried out by randomly resampling the momenta. By tuning the discretization (Δ*s*) and lag time (Δ*t*) parameters, the transition probability matrix becomes essentially tridiagonal (Figure S4). Moreover, it is possible to set up a simple validation test by exploiting the detailed balance condition. Assuming the process is Markovian and reversible, the detailed balance requires *P*_*i*_*R*_*ji*_ – *P*_*j*_*R*_*ij*_ = 0 at equilibrium. Then, the extent by which this relation differs from zero provides an uncertainty measure of the diffusion. In particular, taking into account the detailed balance, eq 9 can be rewritten as
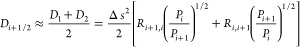
10In the ideal scenario in which the detailed balance strictly holds true, the first and second terms of the r.h.s. of eq 10 do correspond exactly, and eq 9 is retrieved. In real cases, however, the small observed difference between the two terms, purposely renamed *D*_1_ and *D*_2_, is used to provide an estimate of the error of *D* as .

### 2.5 Committor Analysis

In order to assess the reliability of the *s* collective variable (eq 1) as a proper reaction coordinate for describing the ion–water coordination dynamics, we carried out the analysis of the committor as originally proposed in ref (37) and tested in various subsequent works^46−49^ (see also ref (38) for a detailed discussion on the significance and reliability of a reaction coordinate). The committor, π_*i*_(*s*_0_), is defined as the probability for the system to end up in state *s*_*i*_ while starting from a given coordinate *s*_0_, which is usually considered at an intermediate “transition state” point between two or more thermodynamic states. In our monodimensional stochastic model, this function can be expressed as the probability for the system to reach first, and at a later time, the state located on the right side (R) or the left side (L) of the starting coordinate *s*_0_ (the exact time not being relevant)
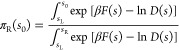
11where π_R_(*s*_0_) is the right committor, that is the probability of a trajectory to reach the state on the right (R) before the one on the left (L) when starting at the top of the dividing barrier *s*_0_. The analysis of the distribution of the committor values *p*(π_R_), typically constructed as an histogram, was evaluated from multiple MD simulations starting from system configurations lying at the separatrix (i.e., π_R_(*s*_0_) = 0.5) between two adjacent coordination states. In practice, the starting configurations (about 1200 selected configurations) in close proximity to a given energy barrier top, *s*_0_ (i.e., *s*_*i*_ < *s*_0_ < *s*_*i*+1_), were generated by the meta-MD simulation. From each of these configurations, 100 replica MD simulations were carried out by resampling randomly the system velocities for about 20 ps (a time interval sufficient to reach the bottom of either left or right coordination states). The obtained collection of ending states (i.e., *s*_L_ or *s*_R_) was then used to estimate the committor probability distribution.

### 2.6 Simulation Details

MD and meta-MD simulations of the five ion–water systems (Ca^2+^, Zn^2+^, Hg^2+^, and Cd^2+^) were carried out to estimate the free energy Δ*F*(*s*) along the coordinate *s*. In each case, a divalent cation was initially placed in a cubic box (40 Å × 40 Å × 40 Å, 2160 water molecules) and solvated with either the TIP3P^50^ (Zn^2+^, Hg^2+^, and Cd^2+^) or SPC/E^51^ (Ca^2+^) water model. In the case of Hg^2+^, a solution at a higher (0.5 M) concentration was also investigated. Every system was neutralized with Cl^–^ counterions. The CHARMM27^50^ force field was used for Hg^2+^, Cd^2+^, and Zn^2+^, while GROMOS35A6^52^ was adopted for Ca^2+^. For Zn^2+^, Hg^2+^, and Cd^2+^, the nonbonded Lennard-Jones potential was modified by adding a 1/*r*^4^ term (i.e., using the so-called 12-6-4 potential developed by Merz and collaborators^15^) to better estimate the charge-induced dipole interactions in M(II) ions. In the dilute solution models, a distance restraint potential was applied between the cation and the counterions to avoid the formation of ionic clusters during the MD simulations, so as to reproduce correctly the ion–oxygen distances in the first solvation shell and the average coordination number as reported in previous studies without counterions.^53,54^ The GROMACS^55^ software package was used to perform a 1000 step minimization, followed by an equilibration (1 ns) in the *NpT* ensemble (at 300 K and 1 atm) to correctly resize the box volume. 1 microsecond MD production runs were performed according to the *NVT* ensemble. Metadynamics^26^ was employed to efficiently obtained the free-energy profile, Δ*F*(*s*), of ion coordination (as described in ref (22)). As a further test, the latter was compared with the one obtained from the corresponding pure MD simulation. Gaussian kernels were added every 5 ps with σ = 0.01 and *h* = 0.1 kJ/mol. The coordinate *s* was recorded at every timestep during both pure MD and metadynamics, and the free-energy profile was successively reconstructed as *F*(*s*) = −*k*_B_*T* ln *P*(*s*), with *P*(*s*) as the observed probability distribution. Standard deviation for *F*(*s*) computed through meta-MD simulations is 1 kJ/mol. Metadynamics simulations were carried out using the open-source, community-developed PLUMED library (ver. 2.6).^56^ LD simulations were carried out by numerical integration of eq 3 with the Euler–Maruyama algorithm.^57^ The integration timestep was set to 2 fs, and for each system, about 1000 replica simulations were performed starting from each state configuration, so as to collect enough statistics for the evaluation of the MFPT.

## 3 Results and Discussion

### 3.1 Assessment of the Kinetic Model

**Figure 3:**
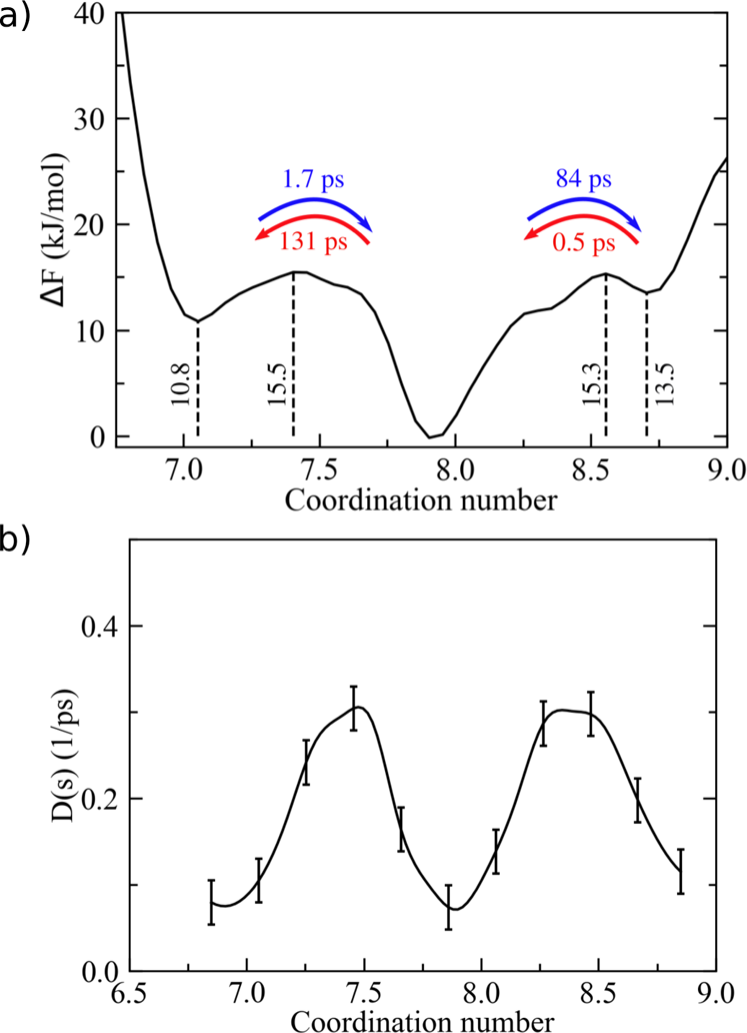
(a) Free-energy landscape of Ca^2+^ coordination in water. Δ*F* values at relevant points (i.e., local minima/maxima) are reported explicitly. MFPTs corresponding to transitions between adjacent states are also reported as computed from the integration of the FP equation. Standard deviation on *F*(*s*) is 1 kJ/mol. (b) Position-dependent diffusion coefficient as a function of the coordination number. Error bars correspond to ± δ*D* (see Section 2.4).

The stochastic kinetic model and the proposed computational procedure to evaluate water exchange rates in the first solvation shell around hydrated ions were tested on a number of different systems, namely, Ca^2+^, Zn^2+^, Hg^2+^, and Cd^2+^. First, we considered the calcium ion since it is known that water exchange is relatively fast around Ca^2+^ and, then, readily accessible to standard MD simulations. The free-energy profile of ion coordination was obtained by both MD and meta-MD simulations, where the latter was carried out following the methodology originally proposed in ref (22) (see details in the Theory and Methods section). Results are reported in Figure S2a showing a very good agreement between pure MD and meta-MD, in line with our previous study,^22^ thus supporting the use of meta-MD to obtain the free energy as a function of the coordination number. In particular, Ca^2+^ displays three ion–water configurations within 15 kJ/mol (Figure 3a), with coordination numbers 7, 8, and 9. The free-energy barrier from coordination 8, which is the most favorable configuration, to 7 or 9 is about 15 kJ/mol, while the barrier to go back to 8 from the other coordination numbers is significantly smaller (<2 kJ/mol). Then, we set out to evaluate the MFPTs for the corresponding coordination state transitions. The position-dependent diffusion coefficient was computed using the computational procedure described in Section 2.4, as depicted in Figure 3b. *D*(*s*) fluctuates between 0.33 and 0.06 ps^–1^ in the relevant space interval (*s* = 7–9), and the corresponding statistical error is on average rather small (0.04 ps^–1^). The resulting MFPTs obtained from our kinetic model using either LD or FP integration are in very good agreement with the ones from long MD simulations, as shown in Table 1. Overall, the MFPTs reflected the observed *F*(*s*) profile (Figure 3a), with τ_7/9→8_ being about 1 ps and τ_8→7/9_ = 80–130 ps, but the kinetic model captured fairly well the existing difference in the average transition times between 8 → 7 and 8 → 9 (Δτ ≈ 45 ps). The latter finding could not have been predicted from the free-energy profile alone and, therefore, highlights the beneficial use of such a kinetic analysis to unravel subtle differences in water exchange dynamics in the first solvation shell. Note that the relatively easy water exchange observed in the case of Ca^2+^ is well in line with both previous quantum mechanical calculations, X-ray and neutron diffraction experiments on CaCl_2_ solutions, and X-ray crystal structures reporting large variations in the coordination number, with values ranging from 6 to 10 (see, e.g., ref (58)).

**Table 1:** MFPT for Ion Coordination in Water, Computed from MD Simulation, LD, and FP Integration (See Section 2.3 for Details)

ion	transition	MD (ps)	LD (ps)	FP (ps)
Ca^2+^	7 → 8	1.58 ± 0.08	1.7 ± 0.1	1.65 ± 0.25
	8 → 9	76 ± 2	88 ± 15	84 ± 11
	8 → 7	120 ± 3	135 ± 12	131 ± 10
	9 → 8	0.40 ± 0.05	0.5 ± 0.1	0.50 ± 0.05
Zn^2+^	6 → 7	304 ± 10	294 ± 25	287 ± 30
	7 → 6	1.2 ± 0.3	1.5 ± 0.3	1.3 ± 0.3
Hg^2+^	7 → 8	0.5 ± 0.2	0.7 ± 0.1	0.7 ± 0.1
	8 → 9	16.8 ± 0.5	21 ± 2	20 ± 3
	8 → 7	27 ± 10 × 10^3^	20 ± 3 × 10^3^	18 ± 3 × 10^3^
	9 → 8	1.70 ± 0.15	1.5 ± 0.3	1.7 ± 0.2
Cd^2+^	6 → 7	1.6^a^	1.8 ± 0.4	2.0 ± 0.5
	7 → 8	2.6 ± 0.2	2.9 ± 0.3	2.8 ± 0.4
	7 → 6	∼10^3^^a^	14 ± 4 × 10^3^	14 ± 3 × 10^3^
	8 → 7	17.7 ± 1.2	18.5 ± 2.0	17 ± 2

a Estimate obtained from the average of the observed transition times.

**Figure 4:**
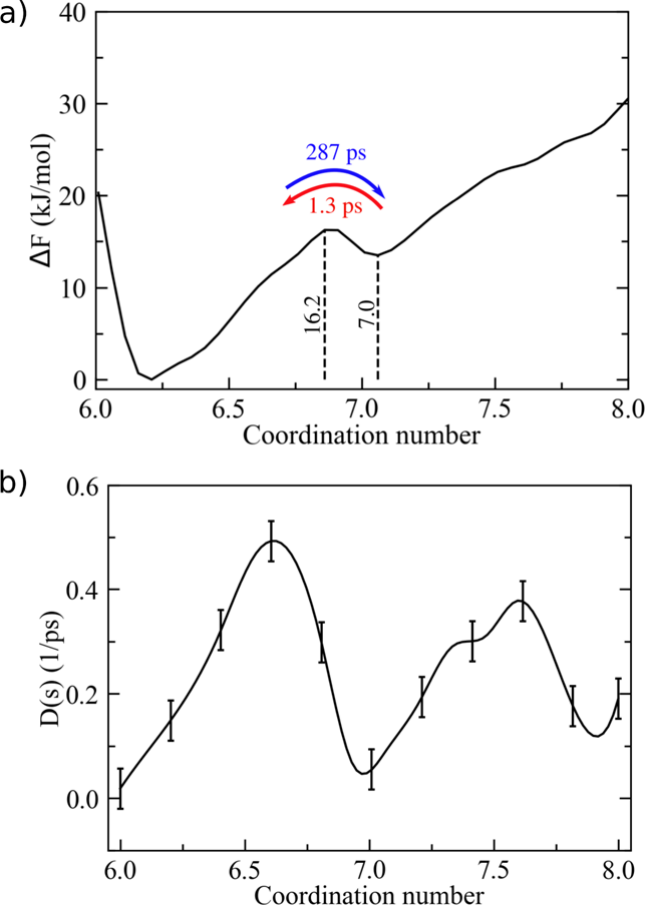
(a) Free-energy landscape of Zn^2+^ coordination in water. Δ*F* values at relevant points (i.e., local minima/maxima) are reported explicitly. MFPTs corresponding to transitions between adjacent states are also reported as computed from the integration of the FP equation. Standard deviation on *F*(*s*) is 1 kJ/mol. (b) Position-dependent diffusion coefficient as a function of the coordination number. Error bars correspond to ± δ*D* (see Section 2.4).

Going to Zn^2+^, we found two free-energy minima (Figure 4a) corresponding to coordination numbers 6 and 7, separated by a relatively low energy barrier (16 kJ/mol) that allowed the sampling of numerous coordination state transitions from standard 1 μs MD simulation. Note that in this case, the free-energy profile, for the chosen force field, clearly pointed toward an associative mechanism as the preferred one for water exchange around the zinc ion. The corresponding MFPTs provided τ_6→7_ ≈ 300 ps and τ_7→6_ ≈ 1 ps, again showing a nice match between our kinetic model and pure MD results (Table 1).

### 3.2 Predicting Water Exchange Rates

**Figure 5:**
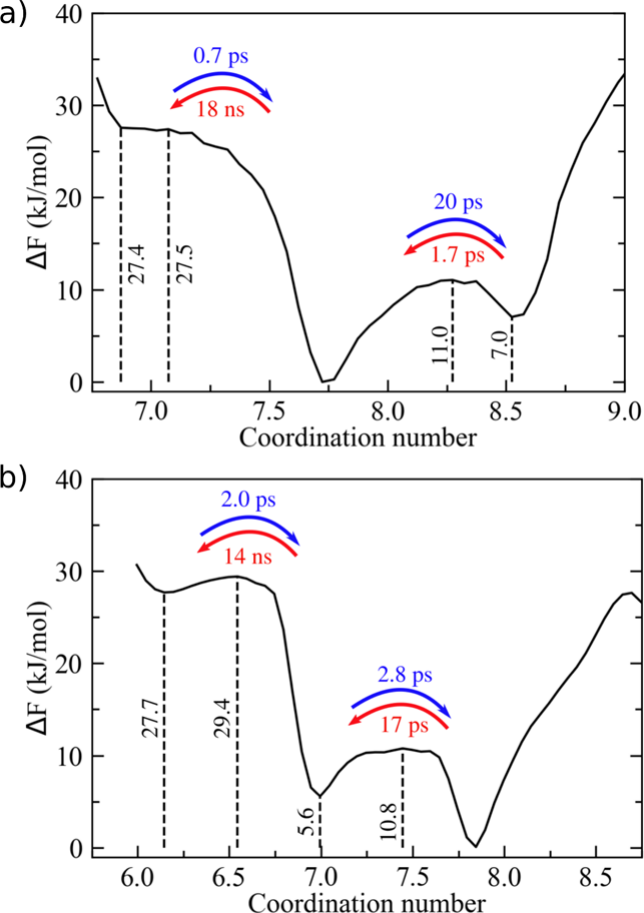
Free-energy landscape of (a) Hg^2+^ and (b) Cd^2+^ coordination in aqueous solution. Vertical dashed lines indicate energy barriers (local maxima) and stable states (local minima) of interest. MFPTs computed from the integration of the FP equation are also reported as insets. Standard deviation on *F*(*s*) is 1 kJ/mol.

The proposed kinetic approach was then applied to a few cations showing high free-energy barriers (>25 kJ/mol) and, hence, “rare” water exchange events not readily accessible to pure MD simulations. For Hg^2+^, we observed three main coordination states, namely, 7, 8, and 9, where the former was rather unfavorable being less stable by about 27 kJ/mol with respect to state 8 (Figure 5a). In this case, of the two possible routes leading to water exchange in the first coordination shell (8 ⇌ 7 and 8 ⇌ 9), only the one based on the associative mechanism appeared feasible. Accordingly, from our 1 μs MD simulation, only 32 transitions from the most probable configuration (i.e., 8) to state 7 were observed, while the number of 8 → 9 transitions was 3 orders of magnitude greater. As a result of the poor statistics, the MFPT of the 8 → 7 transition could not be reliably obtained from the standard MD simulation (i.e., σ(τ) = 10 × 10^3^ ps, Table 1). On the other hand, upon evaluation of the position-dependent diffusion coefficient (Figure S5a) from multiple short MD simulations according to our stochastic model, it was possible to estimate satisfactorily τ(8 → 7) at an affordable computational cost (note that accuracy can be systematically improved if required). In particular, we compared favorably the result issuing from the direct backward Kolmogorov–Chapman equation (eq 5), which is in our view the method of choice, to the alternative methods provided by the integration of the FP equation and LD, as reported in Table S1. As expected, all stochastic approaches provided consistent results, (τ = 18 ± 3 ns). The MFPT evaluated via Kramers equation (Table S1) for the same transition, however, appeared somewhat underestimated (τ = 12.8 ± 2 ns), likely due to the underlying approximations discussed above (Section 2.3). For all other τ's, easily evaluated by the pure MD simulation (i.e., τ ≈ 1–20 ps), the results matched well with the ones of the present kinetic model (Table S1), as for the previously considered cations.

Similarly, we tested the predictive capability of our kinetic model toward Cd^2+^. Three distinct coordination number states emerged from our meta-MD simulation (i.e., 6, 7, and 8 in Figure 5b), among which the octa-coordinated water configuration resulted in the most thermodynamically stable and the hexa-coordinated one the least populated with a separating barrier of about 29 kJ/mol. As a consequence, the observed number of transitions to the latter state was extremely small (i.e., 12), and a direct estimate of the MFPT from the MD simulation was rather problematic providing roughly the order of magnitude of τ (∼ns, Table 1). In this case, the advantage of the stochastic approach proposed in this work was apparent in comparison to the poor statistics affecting the extended MD simulations. For the challenging 7 → 6 transition, the kinetic model provided a τ of about 14 ns, while the other transition times resulted in at least 3 orders of magnitude smaller and in very good agreement with directly observed MD results (Table 1).

It should be pointed out that a close comparison with experiments was not carried out in the present study since this would require a careful consideration of the variety of systems and physico-chemical conditions (e.g., ionic concentration, use of other ligands, temperature, etc.) at which the experiments are typically performed. Nevertheless, we observe that the range of the computed water exchange times for the systems under scrutiny (from ps to ns) is well within the findings issued from past NMR relaxation experiments.^11,29^

### 3.3 On the Relationship between Diffusion and Free Energy

While the position-dependent diffusion coefficient *D*(*s*) and the free-energy function *F*(*s*) do appear as distinct terms of the present stochastic model and, from the computational viewpoint, are independently obtained before being plugged into the FP equation, it is worth noting that their mutual relationship in a real physical system is significant and should not be overlooked. To better investigate this point, we performed a test simulation of the Hg^2+^ system, as seen above, by applying a bias potential equivalent to the one computed from the meta-MD simulation (i.e., the negative of the free-energy profile along the *s* coordinate, −Δ*F*(*s*), see Figure S6), so as to effectively obtain a barrier-less water exchange process. The idea was to inspect the change in *D*(*s*) as a consequence of a significant modification of Δ*F*(*s*), thus highlighting the existing relation between the two ingredients of the kinetic model. In particular, upon applying the bias potential, the system was set free to move between different coordination states (Figure S7a). Under such artificial conditions, the resulting diffusion became basically constant (∼0.1 ps^–1^) throughout the coordinate number space (Figure S7b), a signature of a purely diffusive regime, in stark contrast to the original unbiased system. This finding, in our view, represents a useful warning for those methodologies aiming at obtaining dynamical information from purposely biased systems.

### 3.4 Validation of the Coordination Number as a Reaction Coordinate

**Figure 6:**
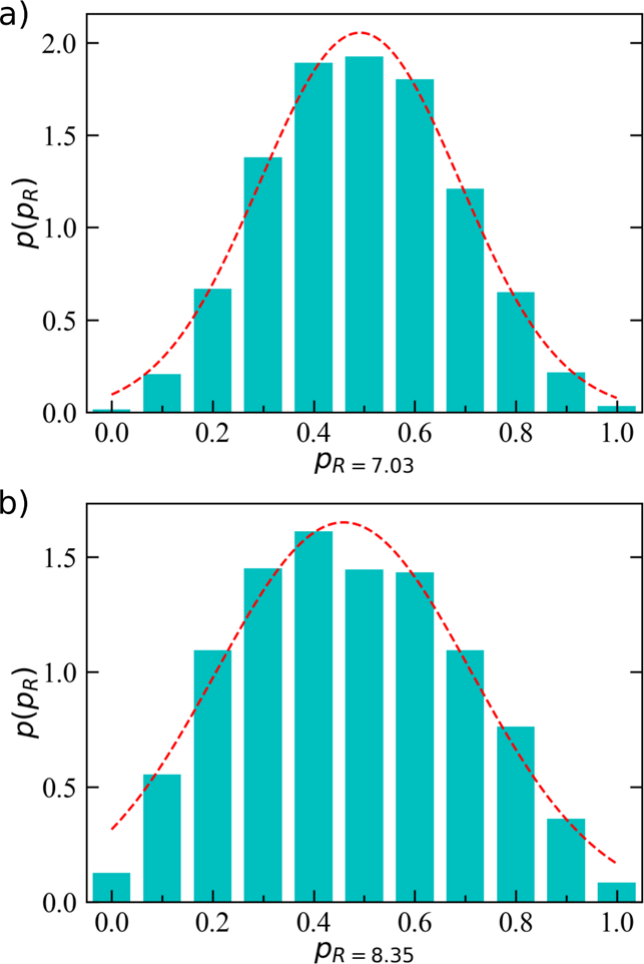
Committor probability distribution for Hg^2+^ coordination in water computed from an ensemble of short MD simulations. 1200 starting configurations were taken at the (a) *s* = 7.03 and (b) *s* = 8.35 barrier top. Then, 100 replica simulations were carried out for each configuration. A Gaussian fit of the probability distribution is also provided (red dashed line).

The Hg^2+^ in the water system was also adopted to validate the use of the coordination number, as defined in eq 1, as a suitable reaction coordinate for the description of the water exchange process. As thoroughly discussed by Peters in a thematic review,^38^ a given collective variable, for example, based on physical considerations or chemical intuition, could prove useful for describing the kinetics of a dynamical transition between two well-defined molecular states without necessarily being an appropriate “reaction coordinate” for the same molecular process, that is not corresponding to the definition of a minimum free-energy pathway and/or not including other relevant coordinates for a proper mechanistic interpretation of the reaction under examination. However, an effective test to assess the quality of a putative coordinate is represented by the committor analysis, as originally proposed in ref (37) (see Section 2.5 for more details). A bell shape distribution of *p*(π_R_) as a function of π(*s*_0_) and peaked around the separatrix region (i.e., π(*s*_0_) = 0.5) is regarded as a positive test for a trial reaction coordinate.^38^ Tests for the committor analysis of the Hg^2+^ system, when considering both free-energy maxima (*p*(*s* = 7.03) and *p*(*s* = 8.35)), were carried out, and the results are depicted in Figure 6. The obtained distributions favorably support the choice of the present coordinate to follow the water exchange process in the first solvation shell around aqua ions.

### 3.5 High Ionic Concentration

**Figure 7:**
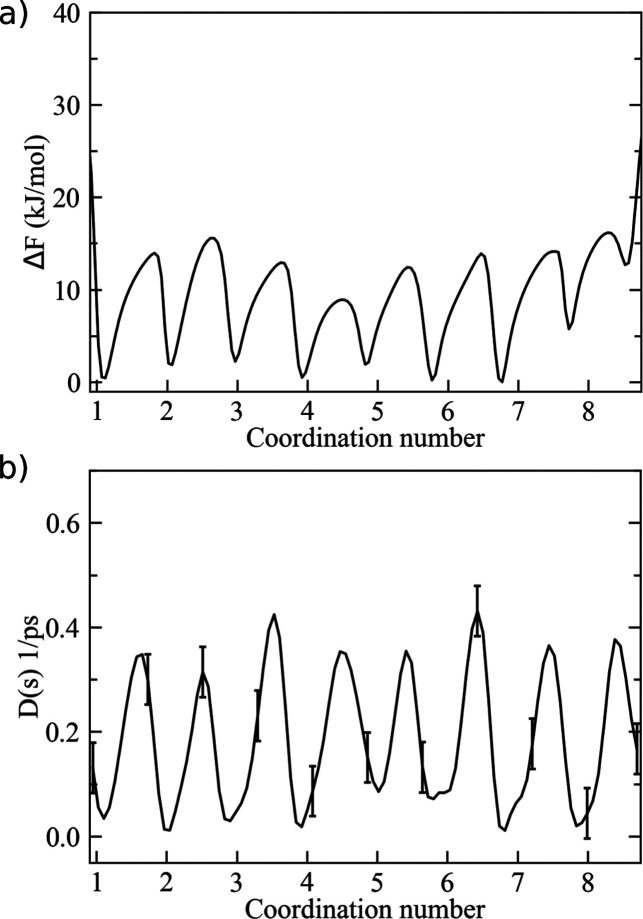
(a) Free-energy landscape of Hg^2+^ coordination in water from 0.5 M HgCl_2_ aqueous solution, as issued from pure MD simulations. (b) Position-dependent diffusion coefficient as a function of the coordination number. Error bars correspond to ± δ*D* (see Section 2.4).

As a further test, we considered a relatively higher concentration (0.5 M) of mercury ions in aqueous solution to assess the robustness of the proposed computational approach under such conditions. First, we observed a noticeable change of the main ion–water configurations in the first solvation shell since a much larger range of coordination numbers around each Hg^2+^ became available (i.e., from 1 to 9, see Figure 7a). In fact, at 0.5 M concentration, ions compete with each other more effectively for acquiring coordinating water molecules, which are now much less abundant with respect to the previous dilute solution. In particular, the effect of the counterions (i.e., Cl^–^) on the first water shell of Hg^2+^ is also greatly enhanced since ionic couples can form (and break apart) more easily at this concentration. As a result, the free-energy landscape of ion coordination showed a noticeable rough surface characterized by multiple local minima (i.e., 9 coordination states) within a limited range of energy (about 10 kJ/mol). Also, dividing energy barriers were significantly reduced to about 10–15 kJ/mol between adjacent coordination states. As a consequence, a single preferential coordination state could not be identified at this concentration. Nonetheless, we again analyzed water exchange dynamics from both pure MD simulations and the kinetic model. In the latter case, we obtained the position-dependent diffusion constant, as depicted in Figure 7b, which overall reflected the same oscillating trend of *F*(*s*). As reported in Table S2, water exchange was observed to occur rather frequently among all states, with MFPTs ranging from ∼10 to ∼80 ps. Once more, the transition times issuing from the stochastic approach revealed, overall, a good agreement with the direct MD estimates, taking into account statistical noise. This finding supported the use of the present computational method for studying ionic solutions at variable concentrations.

## 4 Conclusions

**Figure 8:**
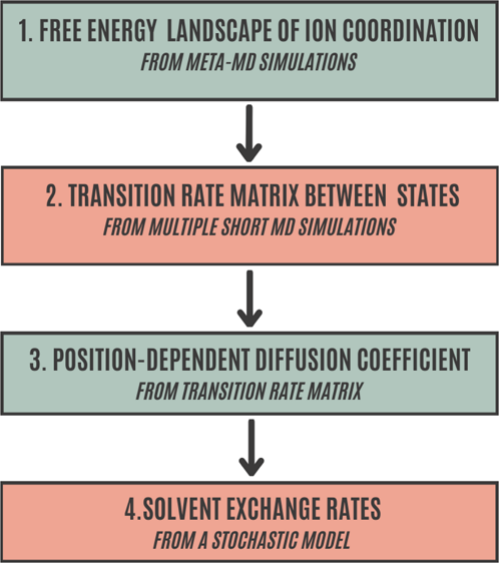
Workflow of the proposed computational protocol to effectively compute ion–water coordination and exchange rates in ionic solutions. A detailed description of the protocol is provided in the text.

In this work, we presented a computational protocol (as sketched in Figure 8) rooted into MD, enhanced sampling, and stochastic methods to obtain a comprehensive picture of solvent coordination and exchange around ions in solution. Our strategy starts from the evaluation of the free-energy landscape as a function of the ion coordination number treated as a continuous collective variable. The free-energy profile provides a “fingerprint” of ion coordination in solution by showing quantitatively the existing complex equilibrium between different solvent coordination states. As a result, the most probable first-shell ion–water configurations, the relative free-energy stability, and the corresponding transition barriers are determined. In a second step, the transition rate matrix describing the dynamical interchange of ion coordination is built up and the position-dependent diffusion constant is evaluated from multiple short MD simulations along the coordination number. At this point, it is worth noting that such a task, the most computationally intensive of our procedure, can benefit from fully independent and parallel MD runs. Then, the computed free energy and diffusion functions are plugged into a FP model to derive the (long-term) time evolution of ion coordination and solvent exchange at timescales not easily accessible to standard MD techniques. Solvent-exchange rates are obtained in terms of MFPTs between coordination states, thus providing a further important observable of ion microsolvation to be compared with available experiments. The computed rates are generally affected by a reasonably small error (within 10–20%), especially in view of the extremely wide range of timescales known from the literature (from 10^–12^ to >10^6^ s). Note, however, that the accuracy of the exchange rate estimates can be improved systematically within the present protocol, while the reliability of the results is closely related to the underlying force field employed. In this regard, we believe that our computational approach can be fruitfully exploited to investigate the agreement between current molecular models and experiments (e.g., NMR relaxation measurements) at an affordable cost. Note that comparison with experiments was not explicitly considered in the present methodological study but will be investigated in future applications. Eventually, this approach can be also employed in force field development, so as to optimize ion–solvent intermolecular potentials toward an additional, usually overlooked, parameter, that is, the solvent-exchange rate.

Notably, the coordination number, adopted in this work as an effective coordinate for monitoring the ion–water coordination, passed successfully the committor analysis test and, therefore, can be regarded as a suitable and physically sound reaction coordinate for the process.^38^ Besides, another advantage of this coordinate is that it is unbiased toward any specific water exchange mechanism in contrast to other coordinates (e.g., the ion–water distance) typically employed in previous computational studies. A further consideration that deserves some comments concerns the assumption of Markovianity. Here, the dynamical process is defined as Markovian, given a suitable discretization of the selected coordinate (i.e., the coordination number), according to the general principle that even a non-Markovian process can turn Markovian at some coarse-grained description (i.e., whenever there is a timescale gap between the relevant coordinate and the other degrees of freedom of the system). In this context, this seems justified by the fact that molecular collisions occur at much faster timescales (∼fs) than the first solvation shell changes (at least ∼ps). Moreover, it is remarkable that exact MFPTs (and rates) can be computed from average transition rates, as obtained using approximate Markovian models, irrespective of the actual distribution of the lifetimes (i.e., the exact non-Markovian trajectory), as discussed in ref (40). In other words, the long-time evolution of the (approximate) stochastic trajectory nicely corresponds, on average, to the detailed MD trajectory, as projected onto the same reaction coordinate.

## Supporting Information Available

Radial distribution functions between ion and water–oxygens; free-energy landscapes of ion coordination; population analysis of the accessible ion coordination states; map of detailed balance condition deviations; diffusion position dependent *D*(*s*) for Hg^2+^ and Cd^2+^; MFPT for Hg^2+^ in water; bias potential applied to the Hg^2+^ system; free-energy landscape and diffusion for the Hg^2+^ system with counteracting potential; MFPT for the 0.5 M HgCl_2_ aqueous solution system; and free-energy landscape of Hg^2+^ from a 0.5 M HgCl_2_ aqueous solution (PDF)
