# Supplementary material for: Stochastic Model of Solvent Exchange in the First Coordination Shell of Aqua Ions
Source: J Chem Theory Comput. 2022 Apr 26;18(5):3164–73. doi: 10.1021/acs.jctc.2c00181 (PMC9097284; doi:10.1021/acs.jctc.2c00181)
Supplement: Supplementary file 1 — ct2c00181_si_001.pdf [file ct2c00181_si_001.pdf]

Supporting Information for "A Stochastic Model of Solvent Exchange in the First Coordination Shell of Aqua Ions"

Luca Sagresti,[†,‡] Lorenzo Peri,[†] Giacomo Ceccarelli,[¶] and Giuseppe Brancato[*,†,‡,§]

†*Scuola Normale Superiore, Piazza dei Cavalieri 7, I-56126 Pisa, Italy*

‡*Istituto Nazionale di Fisica Nucleare(INFN), Largo Pontecorvo 3, I-56127 Pisa, Italy*

¶*Dipartimento di Fisica, Università di Pisa, Largo Bruno Pontecorvo 3, I-56127 Pisa, Italy*

§*Consorzio Interuniversitario per lo Sviluppo dei Sistemi a Grande Interfase (CSGI), Via della Lastruccia 3, I-50019 Sesto Fiorentino (FI), Italy*

E-mail: giuseppe.brancato@sns.it

List of Tables and Figures:

- **Table S1:** MFPT for Hg^{2+} in water.
- **Table S2:** MFPT for the 0.5M $HgCl_2$ aqueous solution.
- **Figure S1:** Radial distribution functions between ion and water.
- **Figure S2:** Free energy landscapes of ion coordination.
- **Figure S3:** Population analysis of the accessible ion coordination states.
- **Figure S4:** Map of detailed balance condition deviations.
- **Figure S5:** Diffusion position dependent D(s) for Hg^{2+} and Cd^{2+}.
- **Figure S6:** Bias potential applied to the Hg^{2+} system.
- **Figure S7:** Free energy landscape and diffusion for Hg^{2+} in water with a counteracting potential.
- **Figure S8:** Free energy landscape of Hg^{2+} from a 0.5M $HgCl_2$ aqueous solution.

Table S1: MFPT for Hg^{2+} coordination in water, computed from long MD simulation (MD), Langevin dynamics (LD), Fokker-Planck integration (FP), Kramers and backward-Kolmogorov (bwKLG) equation (see Methods for details).

	$7 \to 8$ (ps)	$8 \to 9$ (ps)	$8 \to 7$ (ps)	$9 \to 8$ (ps)
MD	0.5 ± 0.2	16.8 ± 0.5	$27 \pm 10 \cdot 10^3$	1.70 ± 0.15
LD	0.7 ± 0.1	21 ± 2	$20 \pm 3 \cdot 10^3$	1.5 ± 0.3
FP	0.7 ± 0.1	20 ± 3	$18 \pm 3 \cdot 10^3$	1.7 ± 0.2
Kramers	0.31 ± 0.06	7.0 ± 0.9	$12.8 \pm 2.0 \cdot 10^3$	0.48 ± 0.08
bwKLG	0.60 ± 0.05	20 ± 2	$17 \pm 3 \cdot 10^3$	1.6 ± 0.3

Table S2: MFPT for 0.5M of Hg^{2+} computed from pure MD simulations and FP integration. In the latter case, MFPTs were estimated using $\Delta F(s)$ from both pure MD (FP) and from meta-MD (FP*) (see Fig. S8).

Transition	MD (ps)	FP (ps)	FP* (ps)
$1 \rightarrow 2$	45 ± 5	71 ± 30	92 ± 38
$2 \rightarrow 1$	17 ± 5	50 ± 30	69 ± 35
$2 \rightarrow 3$	66 ± 20	69 ± 6	36 ± 5
$3 \rightarrow 2$	44 ± 18	67 ± 7	40 ± 5
$3 \rightarrow 4$	19 ± 3	18 ± 4	18 ± 4
$4 \rightarrow 3$	21 ± 9	41 ± 8	9 ± 2
$4 \rightarrow 5$	12 ± 2	11 ± 3	49 ± 18
$5 \rightarrow 4$	11 ± 2	8 ± 2	32 ± 9
$5 \rightarrow 6$	10 ± 2	20 ± 5	30 ± 8
$6 \rightarrow 5$	28 ± 3	29 ± 7	33 ± 8
$6 \rightarrow 7$	19 ± 8	38 ± 10	147 ± 42
$7 \rightarrow 6$	35 ± 15	36 ± 8	21 ± 6
$7 \rightarrow 8$	92 ± 30	71 ± 9	58 ± 14
$8 \rightarrow 7$	2.9 ± 0.4	9 ± 1	15 ± 3
$8 \rightarrow 9$	18 ± 4	32 ± 11	33 ± 8
$9 \rightarrow 8$	1.8 ± 0.4	3.3 ± 0.8	4.0 ± 1.2

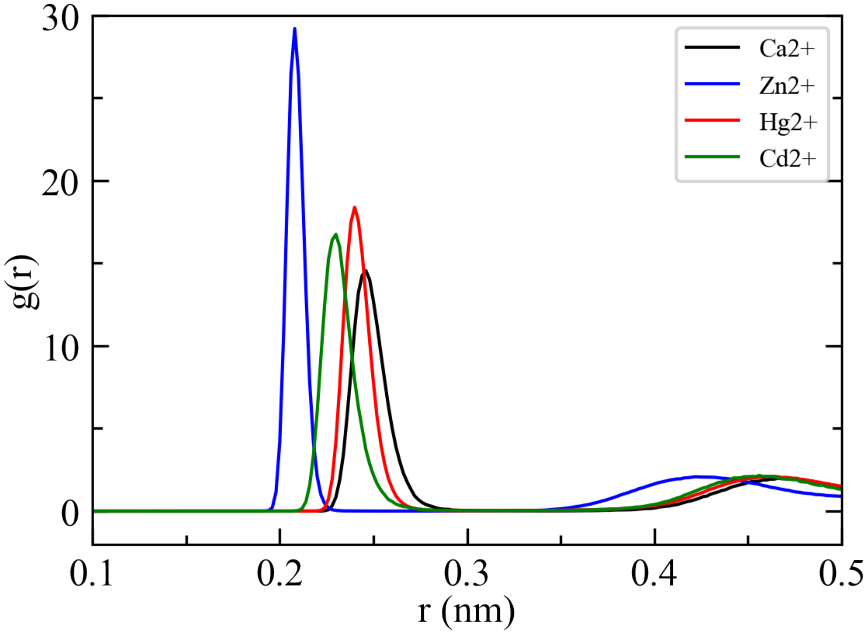

Figure S1: Computed radial distribution function (RDF) of Ca^{2+} (black), Zn^{2+} (blue), Hg^{2+} (red) and Cd^{2+} (green) in water (i.e., Ion-O RDF).

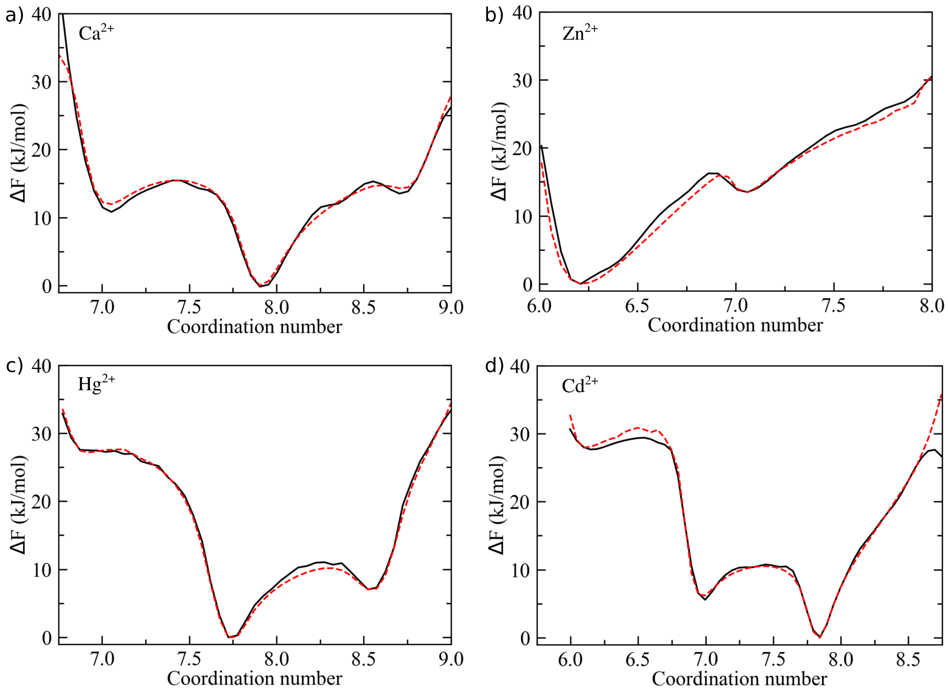

Figure S2: Free energy landscape, $\Delta F(s)$, of ion coordination as issuing from standard MD (red dashed line) and meta-MD (black solid line) simulations for (a) Ca^{2+}, (b) Zn^{2+}, (c) Hg^{2+} and (d) Cd^{2+}.

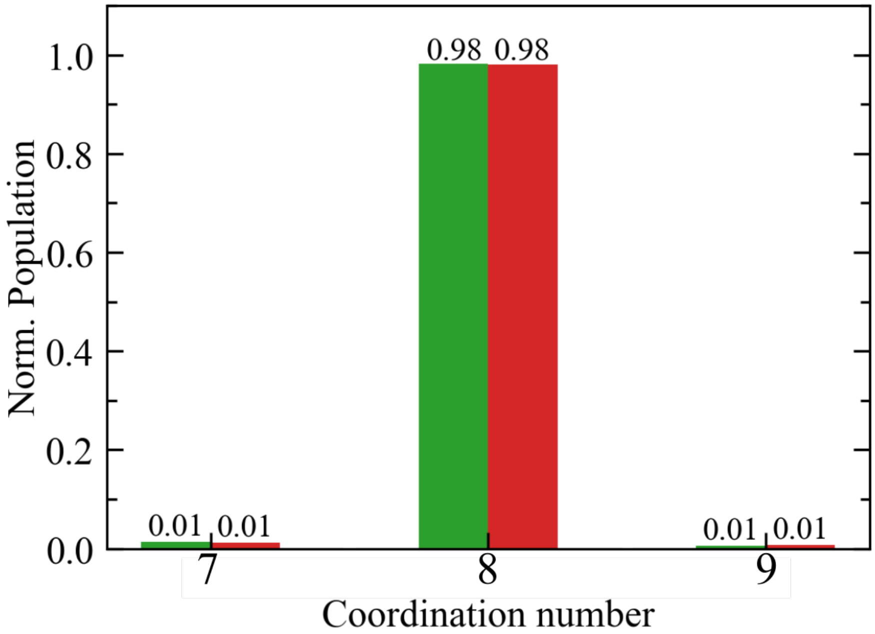

Figure S3: Analysis of the normalized population for each of the three discrete coordination states of Ca^{2+}. Red bars, results computed from the history-based algorithm described in Sec. 2.3. Green bars, results obtained by direct assignment to a coordination state of each sampled MD configuration.

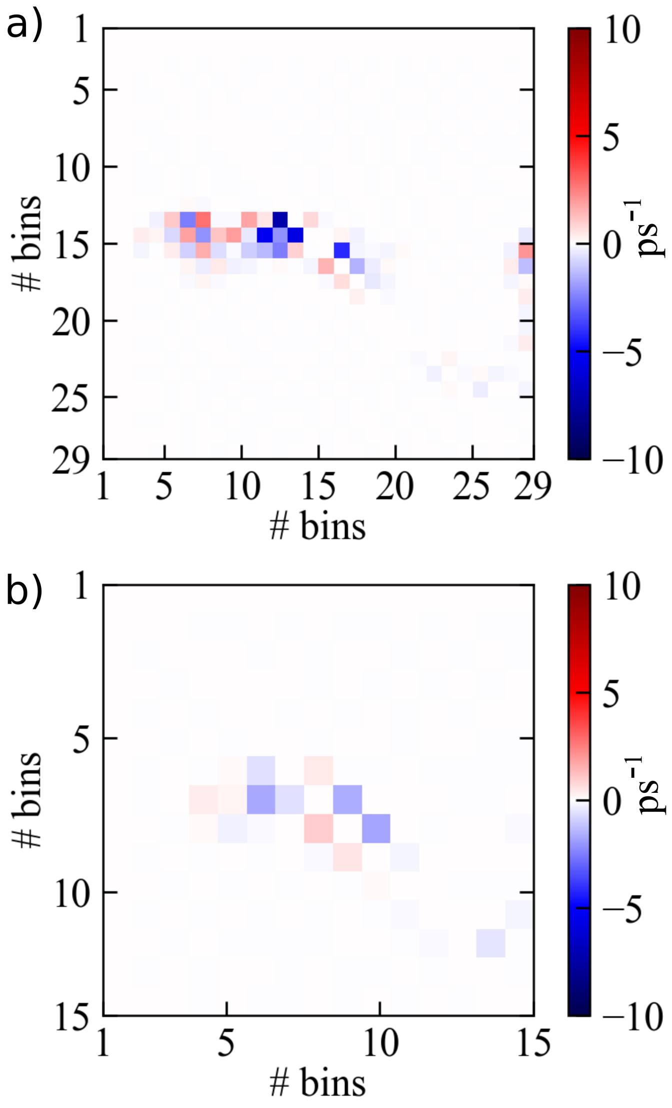

Figure S4: Map of detailed balance deviations ($P_i R_{ji} - P_j R_{ij} \neq 0$). The map highlights how far from the ideal detailed balance condition is the computed transition rate matrix. a) Example of a poor discretization (29 bins along the s coordinate for Hg^{2+}) showing a rough approximation to a birth-death process, around the 13-th bin. b) With 15 bins, the discrepancies are drastically reduced to less than 1 ps^{-1} and then the rate matrix can be accepted to construct $D(s)$. This example shows the importance of a correct discretization of the coordinate s.

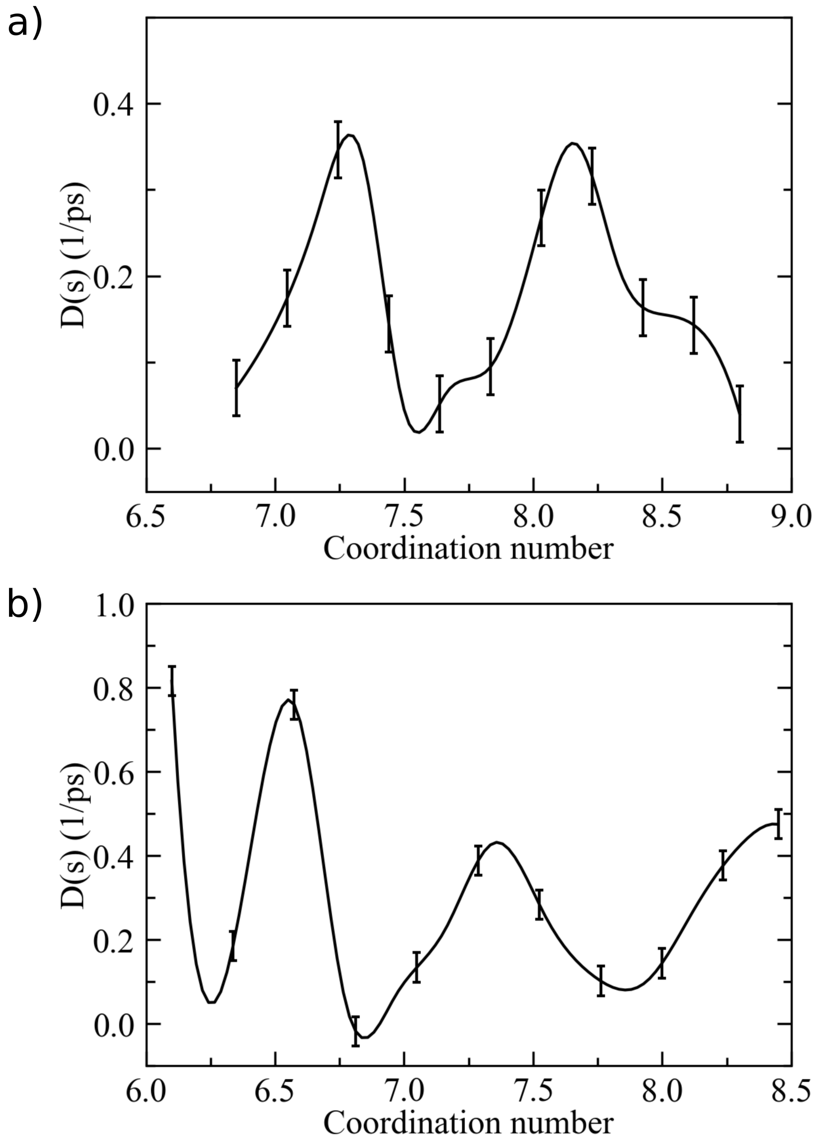

Figure S5: Position-dependent diffusion coefficient, $D(s)$, of a) Hg^{2+} and b) Cd^{2+} ion coordination as computed through the method proposed in Sec. 2.4. Error bars are $\pm\delta D$.

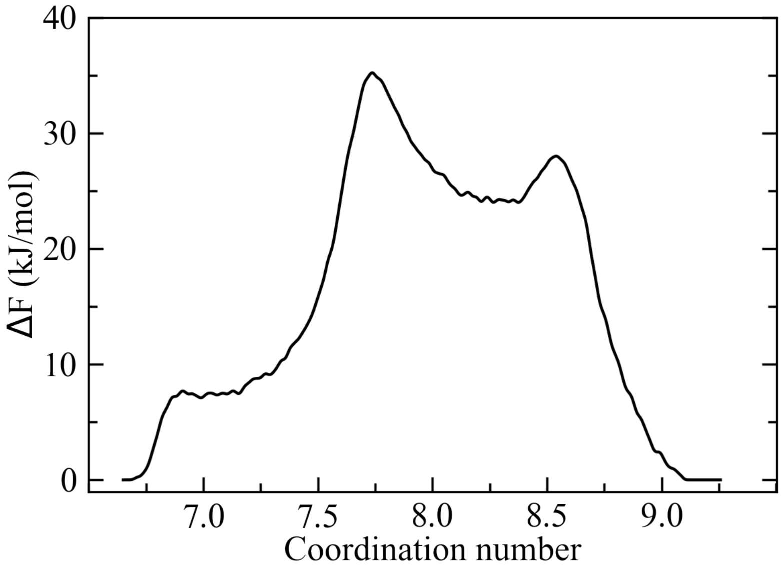

Figure S6: Profile of the bias potential applied to the Hg^{2+} system in a test MD simulation to neutralize any free energy barrier along the water coordination variable, s.

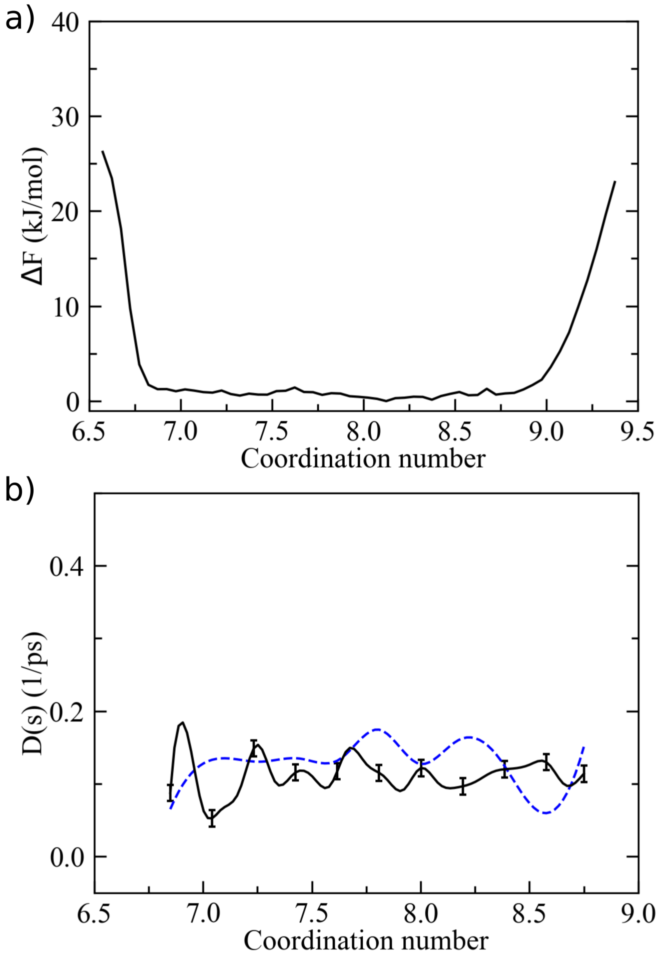

Figure S7: a) Barrier-less free energy profile of Hg^{2+} coordination in water with an applied counteracting potential. b) Position-dependent diffusion coefficient as a function of the coordination number. Error bars correspond to $\pm\delta D$ (see Sec. 2.4). Blue dashed line is the diffusion computed from local mean squared displacements. In both cases, D oscillates slightly around the average value of 0.1 ps^{-1}.

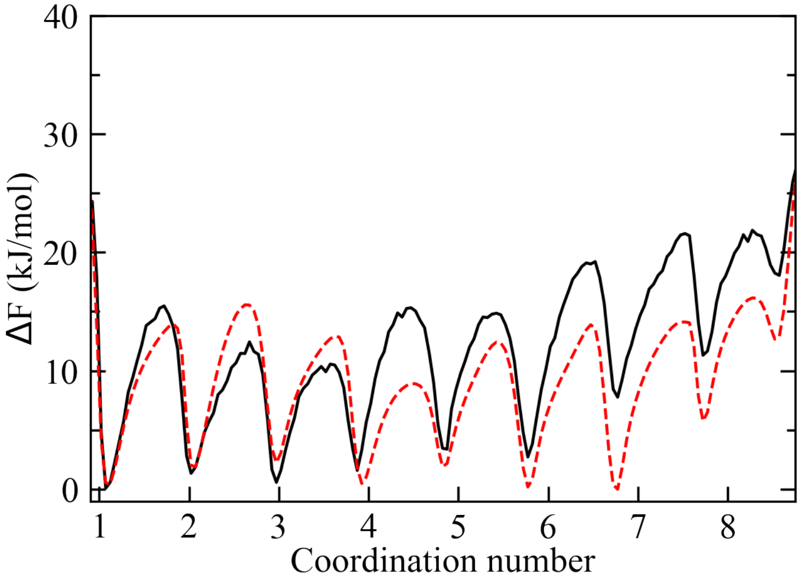

Figure S8: Free energy landscape of Hg^{2+} coordination in water from a 0.5M $HgCl_2$ aqueous solution, as issuing from meta-MD (black solid line) and pure MD (red dashed line) simulations. In this case, the observed deviations should be ascribed to a poorer statistics of the meta-MD simulation, since only one Hg^{2+} ion (out of 20) was considered when computing the bias potential. This can be easily improved using a different implementation of the algorithm, but we preferred to keep the same protocol for consistency with the other meta-MD simulations.
